# PFR peptide, one of the antimicrobial peptides identified from the derivatives of lactoferrin, induces necrosis in leukemia cells

**DOI:** 10.1038/srep20823

**Published:** 2016-02-10

**Authors:** Yan Lu, Teng-Fei Zhang, Yue Shi, Han-Wei Zhou, Qi Chen, Bu-Yun Wei, Xi Wang, Tian-Xin Yang, Y. Eugene Chinn, Jian Kang, Cai-Yun Fu

**Affiliations:** 1 College of Life Sciences, Zhejiang Sci-Tech University, Hangzhou 310018, China; 2 Zhejiang Provincial Key Laboratory of Silkworm Bioreactor and Biomedicine, Hangzhou 310018, China; 3 Department of Oncology, the 117th Hospital of PLA, 14 Lingyin Road, Hangzhou 310013, China; 4 Department of Hematology, Zhejiang Province People’s Hospital, Hangzhou, Zhejiang, China; 5 Institute of Health Sciences, Chinese Academic of Sciences, Shanghai 200031, China; 6 Cancer Signalling Laboratory, Oncogenic Signalling and Growth Control Program, Peter MacCallum Cancer Centre, St Andrews Place, East Melbourne, Victoria 8006, Australia; 7 Key Laboratory of Cell-Based Drug and Applied Technology Development in Zhejiang Province, Hangzhou, People’s Republic of China

## Abstract

LF11-322 (PFWRIRIRR-NH_2_) (PFR peptide), a nine amino acid-residue peptide fragment derived from human lactoferricin, possesses potent cytotoxicity against bacteria. We report here the discovery and characterization of its antitumor activity in leukemia cells. PFR peptide inhibited the proliferation of MEL and HL-60 leukemia cells by inducing cell death in the absence of the classical features of apoptosis, including chromatin condensation, Annexin V staining, Caspase activation and increase of abundance of pro-apoptotic proteins. Instead, necrotic cell death as evidenced by increasing intracellular PI staining and LDH release, inducing membrane disruption and up-regulating intracellular calcium level, was observed following PFR peptide treatment. In addition to necrotic cell death, PFR peptide also induced G_0_/G_1_ cell cycle arrest. Moreover, PFR peptide exhibited favorable antitumor activity and tolerability *in vivo*. These findings thus provide a new clue of antimicrobial peptides as a potential novel therapy for leukemia.

Antimicrobial peptides (AMPs) are cationic molecules generated in the biological defense system as a first line of defense against invading pathogenic microorganisms^1^,^2^. The first human-originated AMP was identified by Zeya and Spitznagel fifty years ago. They discovered the antimicrobial activities of basic proteins in human leukocytes lysosomes^3^. Up to date, the Antimicrobial Peptide Database (APD, http://aps.unmc.edu/AP/main.php) contains 2583 AMPs with a broad spectrum of activities^4^. AMPs are produced by both prokaryotic and eukaryotic organisms and function as a major part of the innate immune defense^5^. AMPs cause cell death either by disrupting the microbial cell membrane integrity, inhibiting extracellular polymer synthesis or impairing intracellular functions^4^,^6^,^7^,^8^. In addition to the antimicrobial activity, a growing number of studies have reported their anticancer activity^9^,^10^.

Among those AMPs, Lactoferrin (LF) is a mammalian cationic iron-binding glycoprotein with a molecular weight of ∼80 kDa. LF is widely distributed in biological fluids and expressed by the immune cells upon pathogen stimulation^11^,^12^,^13^,^14^. LF induces a broad-spectrum of antimicrobial activity against bacteria, fungi, protozoa, viruses and yeasts^15^,^16^,^17^,^18^,^19^, and also possesses antitumor activity^20^,^21^,^22^,^23^,^24^,^25^.

Lactoferricin (LFcin) is released as a potent AMP derived by pepsin digestion of LF^26^. Human LFcin comprises amino acid residues 1–45 of the N-terminus of human Lactoferrin (hLF). LF11, an 11 amino acid residue peptide fragment corresponding to hLFcin 21–31(FQWQRNIRKVR-NH_2_), has weak antibacterial activity^27^,^28^,^29^, and thus, approximately 150 LF11 mutants were designed to optimize its biological activity. These mutant peptides enhance the interaction with target cell membranes and consequently generate potent antimicrobial and endotoxin-neutralizing effects, while having no or little antigenic or toxic effect on normal human cells^30^.

LF11-322 (PFWRIRIRR-NH_2_), also named as PFR peptide or PR peptide^31^,^32^,^33^, is one of LF11 mutants with enhanced cytotoxic activity against *Escherichia coli* (up to 30-fold compared to LF11)^30^. We herein investigated and characterized anti-tumor activity of PFR peptide in leukemia cells.

## Materials and Methods

### Cell culture

Three leukemia cell lines, including murine erythroleukemia (MEL) cells, human promyelocytic leukemia HL-60 cells and human immortalized myelogenous leukemia K562 cells were obtained from Chinese Academy of Medical Sciences & Peking Union Medical College (generous gifts from Professor Jingbo Zhang). The MEL cells and K562 cells were cultured in DMEM (Life Technologies, Carlsbad, USA) and HL-60 cells cultured in RPMI-1640 (Life Technologies, Carlsbad, USA) supplemented with 10% heat-inactivated fetal bovine serum (Sijiqing Biotechnology Co., China) at 37 °C in a humidified atmosphere at 5% CO_2_.

The bone marrow cells were harvested and cultured as described previously^34^. Briefly, BALB/c mice (20 g ± 2 g) were soaked in 75% ethanol for 1–2 min to prevent hair float in the sky. Femurs and tibias were removed from mice and the bone marrow cells flushed from mice femurs and tibias were cultured in IMDM (Life Technologies, Carlsbad, USA) containing10% fetal calf serum (Sijiqing Biotechnology Co., China) and glutamine 2 mM (Lonza, Walkersville, MD, USA) and penicillin/streptomycin(50 U/ml and 50 mg/ml, respectively; Life Technologies, Carlsbad, USA) at 37 °C in 5% CO_2_.

### Drug Treatment

The antimicrobial peptide PFR (PFWRIRIRR-NH_2_) was synthesized by the solid-phase peptide method and purified by high-performance liquid chromatography to more than 98% in Chinese Peptide Company. PFR peptide was dissolved in phosphate-buffered saline (PBS) to 30 mM. The aliquots were stored at −20 °C and thawed on the day of the experiment.

### Cell Viability Assay

Cells were seeded in a 96-well plate at a density of 3 × 10^3^ cells /well and cultured with PFR peptide at various concentrations or buffer alone at different time points as indicated. Then, 10 μl MTT solution (5 mg/ml, Sigma) was added to each well and incubated at 37 °C in 5% CO_2_ for 4 hours. After centrifugation at 3000 g for 15 minutes, the supernatant was removed and DMSO (dimethyl sulfoxide, Sigma) at the volume of 150 μl was added to dissolve the formazan crystals. The absorbance was measured at 570 nm using a microplate reader (Varioscan Flash, Thermo).

### Determination of hemolytic activity

The effect of PFR peptide on human red blood cells (RBCs) was evaluated by a hemolysis assay^35^. Briefly, 100 μl of fresh peripheral blood from a healthy volunteer was added with 4 μl of heparin (5000 IU/ml) and centrifuged at 2000 rpm for 10 minutes at room temperature. The RBCs were further washed three times with sterile PBS and prepared in 2% (v/v) suspension of erythrocytes in PBS. 50 μl of diluted RBCs were seeded in a 96-well plate with 50 μl of PFR peptide at the concentrations of 10, 30, 50, 100, 150, 225, 300 μM in the experimental groups, with 50 μl of 2% (v/v) Triton X-100 in positive control group, or with 50 μl of PBS in negative control group. After incubation at 37 °C for 1 hour, samples were centrifuged at 2200 rpm for 5 minutes and the absorbance was measured at 405 nm using a microplate reader (Varioscan Flash, Thermo). The percent of hemolysis was calculated as: Hemolysis % = [(Sample absorbance – negative control)/(positive control – negative control)] × 100%.

### Scanning Electron Microscopy

The scanning electron microscopy (SEM) was performed as described previously^34^. Briefly, MEL cells, HL-60 cells or K562 cells were seeded at a density of 1.2 × 10^4^ cells /well in 24-well plates and treated with PFR peptide at various concentrations on a sterilized coverslip placed on the bottom of each well. After 24 hours, the medium were removed and cells were washed twice with PBS and then fixed with 1 ml of 3% glutaraldehyde solution for 2 hours at 4 °C. The excess glutaraldehyde solution was removed and the cells were post-fixed by 2% osmium tetroxide for 2 hours followed by dehydration in ethanol baths with a series of concentrations (50, 70, 80, 90 and 100%, 5 minutes in each bath). After the cells were dried in a freeze-drying apparatus (Alpha 2–4 LD plus, Christ, Osterode, Germany), the samples were sputtered with gold using an ion coater and morphology of the cells was assessed using scanning electron microscope (Hitachi S4800 FESEM, Tokyo, Japan).

### Inverted Fluorescent Microscope

Cells were cultured in 96-well flat bottom plates and treated with PFR peptide at various concentrations as indicated. Propidium iodide (PI, BestBio biotechnologies, Shanghai, China) (10 μg/ml, 50 μl) or Hoechst 33342 (Beyotime Institute of Biotechnology, China; 10 μg/ml, 50 μl) was added. After incubation at room temperature for 5 minutes in the dark, image analysis was performed through an inverted fluorescence microscope (TE2000-U, Nikon, Tokyo, Japan) as described previously^36^,^37^.

### Determination of lactate dehydrogenase release

The amount of lactate dehydrogenase (LDH) in the culture medium was determined as described by Taimor *et al.*^38^. Briefly, 3 × 10^3^ cells /well were plated in a 96-well plate and treated with PFR peptide at various concentrations as indicated. Following treatment, the medium was collected and the amount of LDH released into the medium was quantified using the LDH cytotoxicity assay kit according to the manufacturer’s guidelines (Beyotime Institute of Biotechnology, China). The absorbance was measured with a microplate reader (Varioscan Flash, Thermo) at 490 nm.

### Flow cytometric analysis of cell cycle and apoptosis

Exponential growing cells were plated and incubated with PFR peptide for 24 hours. For cell cycle analysis, cells were harvested and fixed in 70% ice-cold ethanol for 24 hours. Cells were then stained with PI (50 μg/ml PI in 0.1% Triton X-100 and 10 μg/ml DNase-free RNase A) for 30 minutes in dark according to the instruction of cell cycle kit (Kaiji Bio Co., Nanjing, China) before analysis using FACSAria (BD Biosciences, Mountain View, CA, USA). For cell apoptosis analysis, cells were incubated with 500 μl of binding buffer and Annexin-V-FITC 10 μl for 15 minutes and then 5 μl of PI for 5 minutes according to the instruction of cell apoptosis kit (Kaiji Bio Co., Nanjing, China) before analysis using FACSAria.

### Calcium analysis

Cells were plated in cover glass-bottom dish (SPL, Korea) designed for confocal microscopic examination at a density of 4 × 10^5^ cells/dish, and cultured for 24 hours. Measurements of intracellular free calcium levels were performed with Fluo-4 AM. The cells were rinsed three times with the assay buffer (130 mM NaCl, 5 mM KCl, 10 mM HEPES, 8 mM D-glucose, 1.2 mM MgCl_2_, and 1.5 mM CaCl_2_, pH 7.4) and then incubated with organic anion transport inhibitor probenecid (Sigma-Aldrich, St. Louis, MO, 2.5 mM), 1 μM Fluo-4 AM (Invitrogen, San Diego, CA, USA), and 0.1% Pluronic F-127 (Invitrogen, San Diego, CA, USA) in the assay buffer for 60 minutes at 37 °C. Before the measurement, cells were rinsed three times with assay buffer and then intracellular Ca^2+^ concentration was measured by monitoring the fluorescence of individual cells at 525 nm using a laser scanning confocal microscope (Nikon, Inc., Tokyo, Japan). Baseline fluorescence was assessed for 50 seconds before adding PFR peptide (300 μM). Image recording continued for additional 950 seconds after stimulation. Ca^2+^ concentrations were expressed as the average fluorescence intensity of 20 cells/field randomly picked up from at least three fields at each time point. The fluorescence data were analyzed using the EZ-C13.20 Free Viewer software (Nikon) and expressed as means ± SEM of three independent experiments.

### Protein extraction and Western blotting

Cells were washed with ice-cold PBS and lysed in the lysis buffer containing 135 mM sodium chloride, 25 mM α-glycerophosphate, 20 mM Tris, 2 mM ethylenediaminetetraacetic acid (EDTA), 2 mM sodium pyrophosphate, 2 mM dithiothreitol, 1 mM sodiumorthovanadate, 10% glycerol, 1% Triton X-100, 2 mg/ml aprotinin, and 5 mg/ml leupeptin (pH7.5). 20 μg of cell lysates were loaded and separated on a 12% SDS-polyacrylamide gel and electrotransferred to PVDF membranes. The membranes were blocked with 5% non-fat milk in Tris-buffered saline containing 0.5% Tween 20 for 2 hours. The membranes were incubated with the following primary antibodies purchased from Bioworld Technology, Inc, China: Rabbit anti-p15 antibody (bs1b1267), rabbit anti-p16 antibody (bs1b1265), rabbit anti-cdk2 antibody (bs102263), rabbit anti-cyclin B1 antibody (bs101392), rabbit anti-cyclin D1 antibody (bs501741), rabbit anti-cyclin D3 antibody (AP500379), rabbit anti-cyclin E1 antibody (bs101085), rabbit anti-Bcl-2 antibody (bs101511), rabbit anti-Bax antibody (bs102538), rabbit anti-Caspase-3 antibody (bs501518), rabbit anti-Caspase-8 antibody (AP100358), rabbit anti-Caspase-9 antibody (AP100359), rabbit anti-PARP antibody (BS70001), mouse anti-GAPDH antibody (AP0063), rabbit anti-Tubulin-α antibody (BS1699). The secondary antibodies, horseradish peroxidase-conjugated goat anti-rabbit or anti-mouse antibodies, were purchased from Hangzhou HuaAn Biotechnology Co. Ltd (Hangzhou, China). Specific proteins were visualized by chemiluminescence with Western Bright^TM^ ECL kit (Advansta corporation, CA, USA) using a Tanon 5500 chemiluminescence detection system (Tanon, Shanghai, China). Densitometry was performed using Image Quant-TL v2005 software (GE healthcare, Chalfont St. Giles, UK). Protein levels were normalized to that of GAPDH or Tubulin α and expressed as fold change.

### Animal experiments

Female BALB/c nude mice of 5 weeks old were obtained from SLAC company (Shanghai, China). To develop the tumor mouse model, *in vitro* growing MEL cells were harvested, washed with PBS and implanted into the right/left flanks of mice (1 × 10^7^ MEL cells in 200 μl PBS per mouse). When tumors reached 100–150 mm^3^, the mice were divided randomly into three groups (n = 10 mice for each group). For the treatment groups, mice were treated with PFR peptide at a dose of 10 mg/kg via either tail intravenous injection or *in situ* injection once daily for 14 days. PFR peptide was prepared in PBS at 2 mg/ml (w/v) and the dose volume was 5 ml/kg mouse weight. For the control group, the mice were treated with PBS via *in situ* injection. Tumor size was measured by vernier caliper every day, and the body weight for each mouse was monitored. Tumor volume (mm^3^) was calculated using the following formula: V(mm^3^) = L(mm) × W(mm)^2^/2, where L and W were the longest and widest diameter of tumor, respectively. At the end of the experiments, all animals were sacrificed according to institutional guidelines. Tumors were resected and fixed in formalin for paraffin embedding.

All experimental protocols were performed in accordance with instruction guidelines from the China Council on Animal Care and approved by the guidelines of the Ethics Committee of Animal Experiments at Zhejiang Sci-Tech University.

### Statistical Analysis

Each experiment was performed at least three times. Data was expressed as mean ± SEM (standard error of the mean). Statistical significance of differences between means was determined by one-way analysis of variance (ANOVA) followed by the Dunnett test for post hoc multiple comparisons. A value of *p* < 0.05 was considered significant.

## Results

### PFR peptide selectively inhibited cell proliferation in leukemia cells

**Figure 1:**
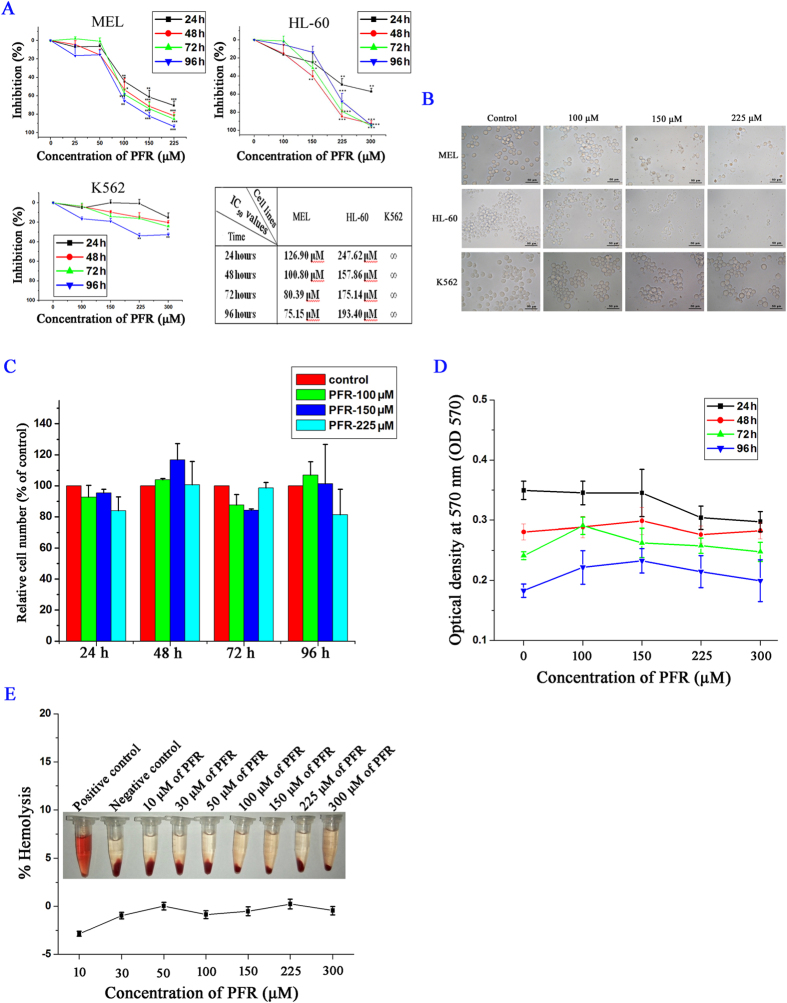
PFR peptide inhibited cell proliferation in leukemia cells. (**A**) MTT assay in three leukemia cell lines MEL, HL-60 and K562 treated with PFR peptide at different dosages, and the IC_50_ values at each time point as indicated (∞ means that it is undetectable) (**B**) The morphological changes after PFR peptide treatment for 72 hours. (**C**) Relative cell number of normal mouse bone marrow cells after exposure to various concentrations of PFR peptide at the indicated time points. (**D**) MTT assay in mouse normal bone marrow cells treated with PFR peptide at different dosages. (**E**) Relative rate of hemolysis in human RBCs upon incubation with PFR peptide at increased concentrations. *p < 0.05, **p < 0.01,***p < 0.001, compared with the control group.

Cell viability in the presence of PFR peptide was assessed by MTT assay in three types of leukemia cell lines, including murine erythroleukemia MEL cells, human promyelocytic leukemia HL-60 cells and human immortalized myelogenous leukemia K562 cells. Both MEL and HL-60 cells showed a dose-dependent reduction of cell viability following PFR peptide treatment with higher sensitivity observed in MEL cells (Fig. 1A). In contrast, K562 cells exhibited a much lower sensitivity to PFR peptide. After treatment with the highest concentration of PFR at 225 μM for 96 hours, K562 cells showed approximately 66% viability compared to 8% and 22% viability in MEL and HL-60 cells, respectively (Fig. 1A). These results suggested that PFR peptide inhibited cell proliferation in certain types of leukemia cells. The morphological assessment after PFR peptide treatment confirmed the MTT results (Fig. 1B). It is noted that there was considerable cellular debris in MEL and HL-60 cells treated with PFR peptide, indicating that PFR peptide induced cell death.

To investigate the potential toxic effect of PFR peptide on normal cells, the bone marrow cells isolated from normal mice were exposed to PFR peptide at a series of doses and cell growth was assessed. There was no significant change in cell number (Fig. 1C) with cells remaining intact and round following the treatment compared with the control cells (Supplementary Figure S1). MTT assay showed no significant inhibition of cell proliferation in the presence of PFR peptide (Fig. 1D). Moreover, no hemolytic toxicity was observed in human RBCs (Fig. 1E). Overall, our data suggested that PFR peptide had little cytotoxicity on normal cells.

### PFR peptide did not induce apoptosis in leukemia cells

**Figure 2:**
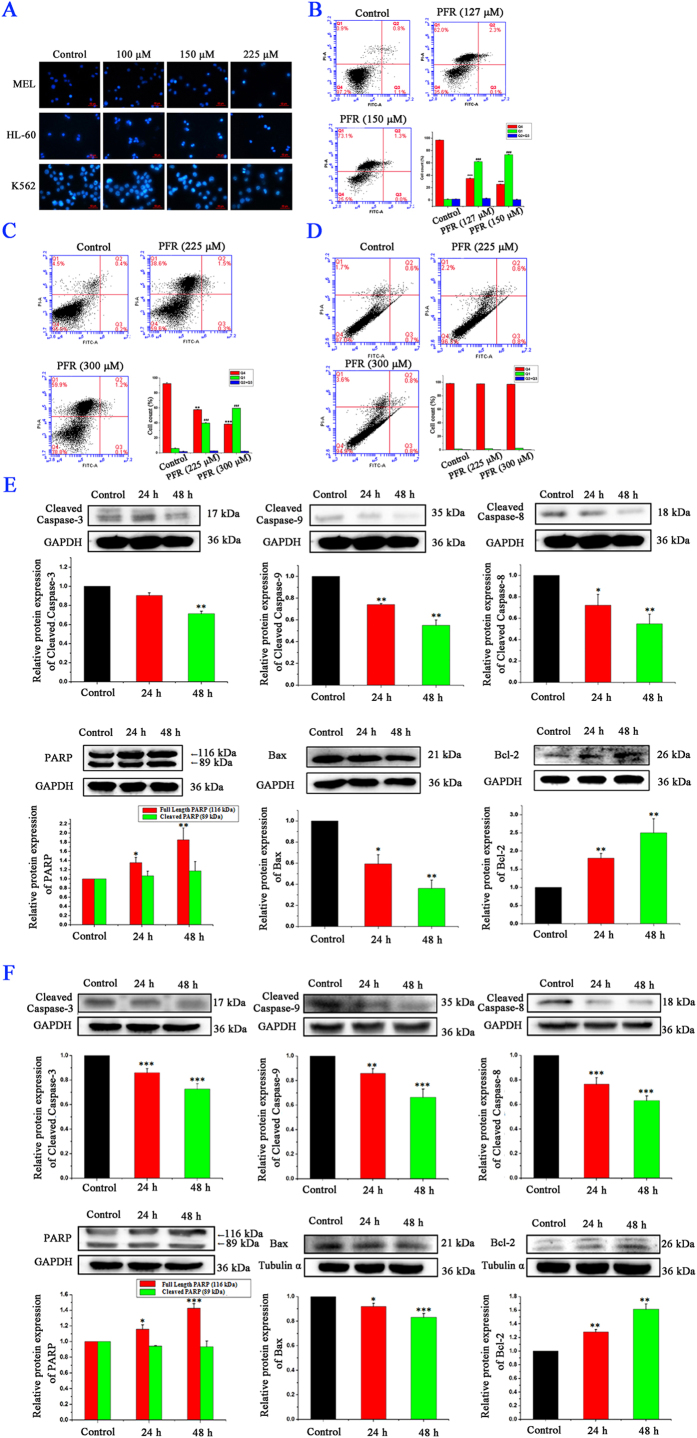
Apoptosis induction did not contribute to the cytotoxic effect of PFR peptide in leukemia cells. (**A**) Fluorescence microscopic analysis of MEL, HL-60 and K562 cells stained Hoechst 33342 after exposure to PFR peptide for 24 hours. (**B–D**) Flow cytometry analysis of leukemia cell lines MEL (**B**), HL-60 (**C**) and K562 (**D**) treated with PFR peptide for 24 hours and stained with Annexin-V-FITC/PI. The proportion of early apoptotic cells (Q3 area), later apoptotic cells (Q2 area) and necrotic cells (Q1 area) was measured in one representative experiments and statistical results from at least three independent experiments. ***p < 0.001, **p < 0.01 compared with the Q4 proportion in control group; ^*###*^*p* < 0.001, compared with the Q1 proportion in control group. (**E**,**F**) Western blotting showing changes of abundance of apoptosis related proteins in MEL cells (**E**) and HL-60 cells (**F**) treated with PFR peptide for 24 and 48 hours (127 μM in MEL cells and 225 μM in HL-60 cells). *p < 0.05, **p < 0.01, ***p < 0.001, compared with the control group.

To explore whether PFR peptide induced apoptotic cell death, cells were stained with Hoechst 33342, a non-toxic specific vital stain for DNA^39^. Interestingly, all three cell lines treated with PFR peptides showed faintly stained nuclei and homogeneously dispersed chromatin as same as the control cells, and did not display typical apoptotic changes such as highly condensed chromatin and small dispersed apoptotic bodies (Fig. 2A). One of the early events of apoptosis includes translocation of membrane phosphatidylserine (PS) from the inner side of the plasma membrane to the surface. Annexin V, a Ca^2+^-dependent phospholipid-binding protein, has a high affinity for PS, and thus fluorochrome-labeled Annexin V can be used for the detection of exposed PS by flow cytometry. Consistently, Annexin V and PI staining assay showed that the proportion of MEL cells distributed in the areas of early apoptosis (Annexin V positive only, Q3 area) and later apoptosis (Annexin V and PI double positive, Q2 area) after exposure to PFR peptide was not significantly changed compared to the control cells. Instead, the proportion of PI stained cells in the Q1 area, which are regarded as necrotic cells (Fig. 2B), was markedly increased after exposure to PFR peptide. Similarly, an increase of the proportion of PI stained cells (Q1 area) upon PFR peptide treatment was also observed in HL-60 cells (Fig. 2C). Nevertheless, there was no significant induction of PI staining in K562 cells (Fig. 2D), consistent with the mild inhibitory effect of PFR peptide on cell proliferation measured by MTT assay (Fig. 1A). The execution of apoptosis requires the activation of the caspases such as caspase 9 and caspase 3, which can be shown by its cleavage and the cleavage of its downstream substrate including PARP. In PFR-treated MEL cells and HL-60 cells, the abundances of cleaved caspase 8, 9 and 3 were all decreased and the expression level of full length of PARP was increased whereas the level of its cleaved form remained unchanged, indicating a lack of activation of apoptotic pathways in both cell lines (Fig. 2E,F). Absence of apoptosis induction was also supported by the decrease of abundance of pro-apoptotic protein Bax and increase of anti-apoptotic protein Bcl-2 expression level (Fig. 2E,F).

Taken together, classical features of apoptosis, including chromatin condensation, loss of membrane asymmetry in the early phases of apoptosis, caspase activation and increase of abundance of pro-apoptotic proteins, were not observed in MEL and HL-60 cells in response to PFR peptide. Therefore, our data is against PFR peptide-induced apoptosis in leukemia cells.

### PFR peptide induced necrosis in leukemia cells

**Figure 3:**
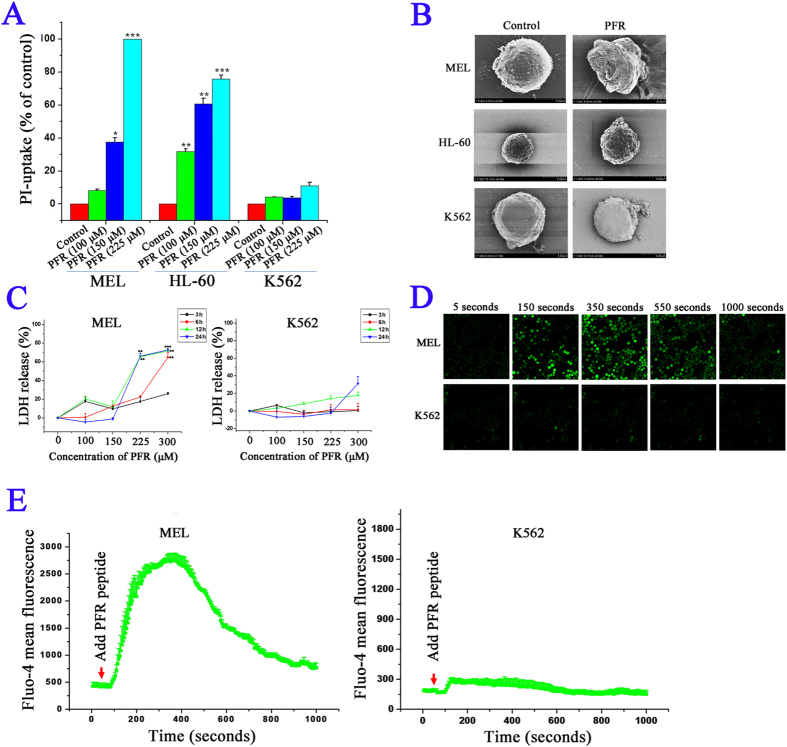
PFR peptide induced necrosis in leukemia cells. (**A**) PI staining analysis of leukemia cell lines MEL, HL-60 and K562 treated with PFR peptide for 24 hours. (**B**) Scanning electron microscopy to assess the morphology of MEL, HL-60 and K562 cells treated with 300 μM of PFR peptide for 24 hours. (**C**) LDH release by MEL and K562 cells after exposure to PFR peptide at the indicated time points. (**D**) Respective images of intracellular fluorogenic calcium indicator Fluo-4 AM staining in MEL cells and K562 cells before and after treatment with PFR peptide. (**E**) Statistical results of Fluo-4 AM fluorescence intensity in MEL and K562 cells from at least three independent assays. *p < 0.05, **p < 0.01, ***p < 0.01, compared with the control group.

Necrosis is another type of cell death in response to insults. Unlike apoptosis, necrotic cells display early rupture of plasma membranes, which can be assessed by PI up-take assay. 24 hours after exposure to PFR peptide, increased cytoplasmic PI staining was detected in both MEL and HL-60 cells in a dose-dependent manner, further confirming the increase of proportion of PI positive cells detected by Annexin V and PI staining assay and suggesting that PFR peptide induced cell membrane disruption (Fig. 3A and Supplementary Figure S2). In contrast, PI staining in K562 cells treated with PFR peptide was not significantly changed, consistent with the MTT results showing refractory to PFR treatment by K562 cells (Fig. 3A and Supplementary Figure S2).

We further performed the scanning electron microscopy to gain more insight into morphological changes induced by PFR peptide. The untreated control cells showed the round shape and smooth surface while PFR treated MEL and HL-60 cells exhibited irregular shape with either corrugated surface or cell swelling (Fig. 3B). Despite the modest inhibitory effect on cell proliferation (Fig. 1A), membrane disruption with protruding bubbles on the surface was also observed in a small proportion of K562 cells (Fig. 3B), in line with a slight increase of PI staining (approximately 10%) after 24 hour treatment with 225 μM of PFR peptide (Fig. 3A). These morphological changes suggest disruption of cell membrane following PFR peptide treatment.

Release of LDH is a biomarker of cell membrane damage and necrotic cell death^40^,^41^. Indeed, PFR peptide produced a dose- and time-dependent increase of LDH release in MEL cells as early as 3 hours after exposure to PFR peptide (Fig. 3C). In contrast, release of LDH in K562 cells in response to PFR peptide was not detected, which was correlated to lack of significant changes on cell membrane permeability measured by PI staining (Fig. 3A).

Increase of intracellular calcium can result in activation of programmed necrosis pathway^42^. We thus further examined the effects of PFR peptides on intracellular calcium concentration. Changes in cytosolic Ca^2+^ level in MEL cells and K562 cells after treatment with PFR peptide were monitored with intracellular fluorogenic calcium indicator Fluo-4 AM. A rapid and dramatic increase of Ca^2+^ level in MEL cells was detected upon addition of PFR peptide (300 μM) at 50 seconds. This up-regulation of intracellular Ca^2+^ content is possibly due to a rapid influx of extracellular Ca^2+^ after loss of plasma membrane integrity or release of Ca^2+^ from intracellular calcium stores. The intracellular Ca^2+^ level reached its peak at approximately 400 seconds and then gradually declined. At 1000 seconds, the cytosolic Ca^2+^ concentration returned to the basal level (Fig. 3D,E). In contrast to the rapid and robust Ca^2+^ response observed in MEL cells, there was no change of intracellular Ca^2+^ level in K562 cells after treatment with PFR peptide (Fig. 3D,E).

All together, PI staining, membrane disruption, LDH release and increase of intracellular Ca^2+^ content suggested that integrity of plasma membrane is disrupted by PFR peptide, further supporting that necrosis, rather than apoptosis, is a major mechanism of leukemia cell death in response to PFR peptide.

### PFR peptide induced cell cycle arrest in leukemia cells

**Figure 4:**
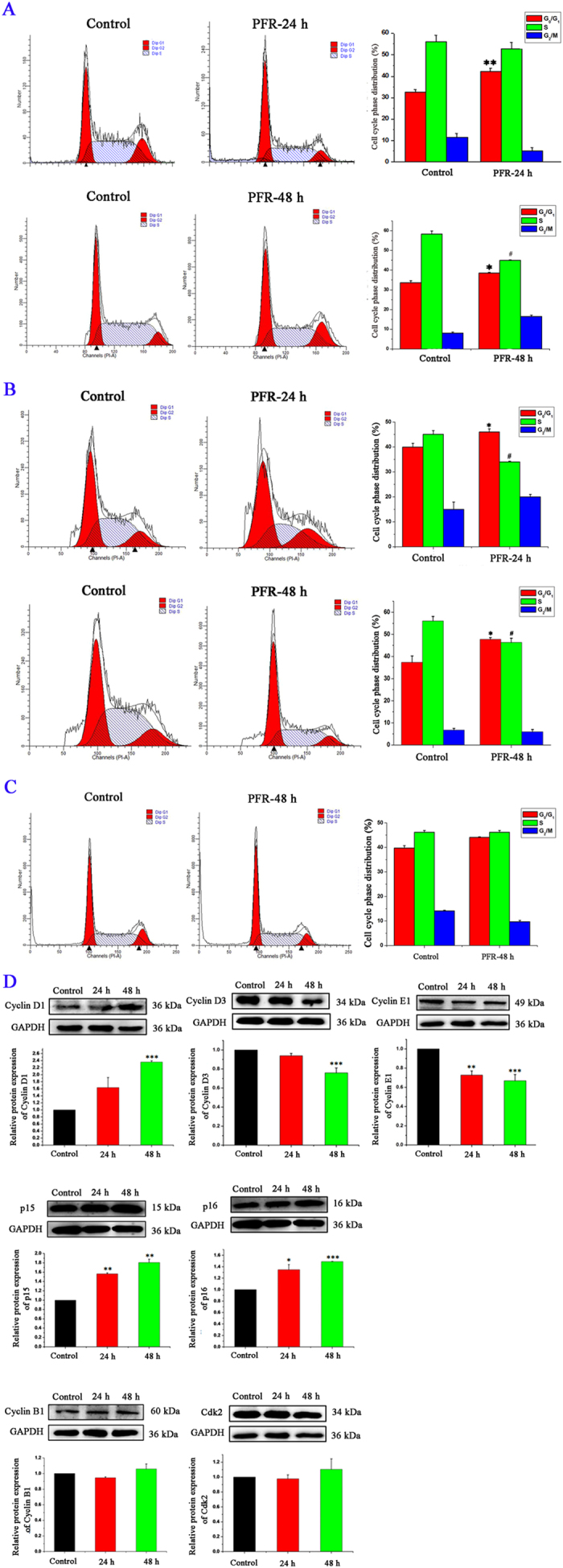
PFR peptide induced cell cycle arrest in leukemia cells. (**A–C**) Cell cycle analysis of leukemia cell lines MEL (**A**), HL-60 (**B**) and K562 (**C**) treated with PFR peptide at 127 μM, 225 μM and 300 μM, respectively, for 24 hours and 48 hours, respectively. *p < 0.05, **p < 0.01, compared with G_0_/G_1_ phase in the control group; #*p* < 0.05, compared with S phase in the control group. (**D**) Western blotting showing changes of abundance of cell cycle related proteins in MEL cells after treatment with PFR peptide at 127 μM for 24 and 48 hours. *p < 0.05, **p < 0.01, ***p < 0.001, compared with the control group.

In addition to necrosis, PFR peptide induced cell cycle arrest in MEL cells as evidenced by significant increase of cell population in G_0_/G_1_ phase after 24 hour treatment and significant decrease of cell population in S phase after 48 hour treatment (Fig. 4A). Similar changes were also observed in HL-60 cells (Fig. 4B) whereas there was no significant change in cell cycle progression in K562 cells treated by PFR peptide (Fig. 4C). Consistently, the protein expression levels of cyclin D3 and cyclin E1 were decreased and CDK4/6 inhibitors p15 and p16 were increased following treatment with PFR peptide in a time-dependent manner (Fig. 4D). CDK2 and cyclin B1, which are activated during S and G2-M phase, respectively, remained unchanged after PFR peptide treatment. Interestingly, the abundance of cyclin D1 was increased after exposure to PFR peptide (Fig. 4D), which is consistent with the potential role of cyclin D1 in modulation of programmed cell death^43^,^44^,^45^,^46^.

### PFR peptide inhibited leukemia cell growth *in vivo*

**Figure 5:**
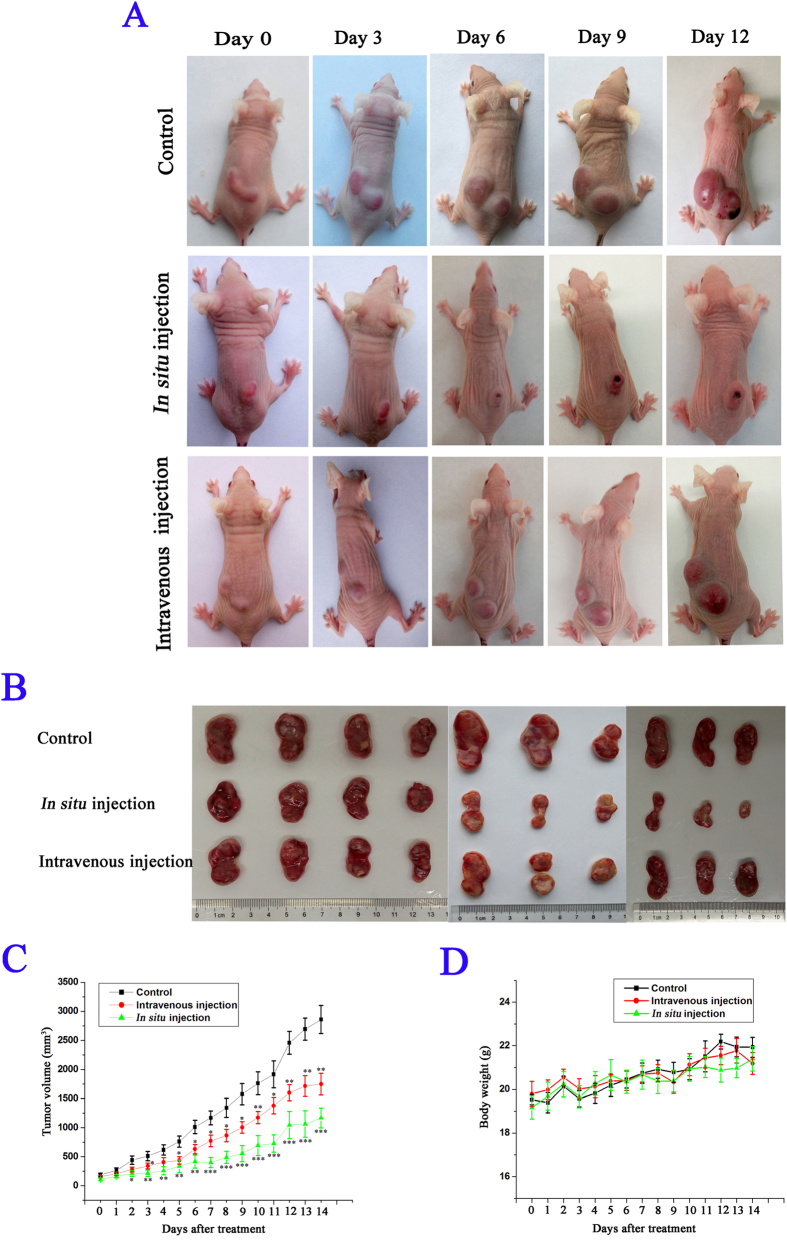
PFR peptide inhibited leukemia cell growth *in vivo*. Nude mice bearing MEL tumors were treated with PFR peptide at dose of 10 mg/kg once daily via either intravenous injection or *in situ* injection. The tumor volume and body weight were measured and calculated as described in Materials and Methods. (**A**) The image of representative mice bearing MEL tumors in each group photographed every three days. (**B**) The image of tumors excised from all mice on day 14 (n = 10 mice for each group). (**C**,**D**) Tumor volume (**C**) and body weight (**D**) measured in MEL mouse model. Bars, SEM; *p < 0.05, **p < 0.01, ***p < 0.001, differs from the corresponding control.

The antitumor activity of PFR peptide was examined in immunocompromised mice transplanted with MEL cells. PFR peptide was administered at 10 mg/kg once daily via intravenous injection or *in situ* injection for 14 days. PFR peptide suppressed tumor growth (Fig. 5A,B), by 39% (P = 0.0019) via intravenous injection and by 59% (P = 0.0004) via *in situ* injection relative to vehicle control at the end of study (Fig. 5C). In this model, PFR peptide was well tolerated with no difference in weight gain between groups (Fig. 5D) and no detectable signs of toxicity, suggesting that this agent may have therapeutic potential in clinic.

## Discussion

Accumulating evidence supports that the short cationic peptides can selectively kill cancer cells with the minimal toxicity to human normal cells, representing a potential novel class of anticancer agents^9^,^10^. PFR peptide, one of the shortest α-helical, amphipathic and arginine-rich AMP to date, was derived from the modified N-terminal domain of human lactoferrin. It causes bacteria cell death by dispersing the membrane components and increasing the membrane permeability^47^. However, the antitumor activity of PFR peptide remains unknown. This study, for the first time, demonstrated that PFR peptide induced necrotic nature of cell death in leukemia cells and inhibited tumor growth in subcutaneously transplanted leukemia mouse model. In addition, we did not observe the toxic effect of PFR peptide on mouse normal bone marrow cells and human whole blood cells, which strengthen the previous finding that PFR peptide did not induce hemolysis in human erythrocytes up to the concentration of 500 μg/ml^33^.

Cell membrane is the primary target site for AMPs. Indeed, the antibacterial activity of PFP in *Escherichia coli* was correlated with the degree of disruption of bacterial membrane mimics^30^. It is becoming evident that the major mechanism of action of cytotoxic effect of AMPs is attributed to electrostatic interaction between the positively charged AMPs and negatively charged lipids on the target membranes and further disruption of the membrane integrity^9^,^31^. PFR peptide is reported to promote segregation of anionic lipids from zwitterionic lipids^32^. Alternatively, certain cationic AMPs (e.g., magainin 2, melittin, and LficinB) have been shown to disrupt plasma membranes via micellization or pore formation in cancer cells^48^,^49^,^22^. We observed disruption of cell membrane morphology, increase of PI-uptake and intracellular LDH release, and elevated intracellular Ca^2+^ content, indicative of an increase of membrane permeability and cell rupture in response to PFR peptide. These morphological and molecular features support necrotic cell death induced by PFR peptide. Moreover, reduction of cell viability measured by MTT assay was correlated to the degree of membrane disruption assessed by these morphological and molecular changes.

Interestingly, several studies reported that LfcinB, the full length form of PFR peptide, induced apoptosis in cancer cells, including THP-1 human monocytic tumor cells^25^, Jurkat T-leukemia cells^22^, human T-leukemia cells^21^ and human neuroblastoma cells^23^. Instead, we did not observe chromatin condensation, Annexin V staining and activation of caspases and its substrate PARP, which argue against apoptotic cell death induced by PFP peptide. This discrepancy may be partially due to (1) the mode of action exerted by PFR peptide, a modified active fragment of LfcinB, different from its full-length form and (2) distinct membrane composition among different cell types.

Moreover, the electrostatic interaction between the positively charged AMPs and the negatively charged components in cell membrane is believed to play a major role in target selectivity and drug specificity. Single intact cells have unique membrane fatty acid and phospholipid composition, and cholesterol content, which affect membrane charge, fluidity as well as cellular functions^50^. Epandet al. reported that the selectivity of PFR peptide against different strains of Gram positive bacteria was related to preferential binding of PFR to acidic phospholipid on bacterial membranes^32^. The membrane composition of tumor cells differs from that of normal cells, for example, the exposure of the negatively charged phospholipid phosphatidylserine (PS) on the outer leaflet of the cancer cell membrane^51^,^52^,^53^,^54^,^55^. In contrast, the outer leaflet of plasma membrane in normal cells exhibits an overall neutral charge due to its main components: the zwitterionic phosphatidylcholine (PC) and sphingomyelin (SM)^56^. Therefore, the unique membrane composition in tumor cells may make them more susceptible to cationic AMP targeting. In addition, the differences in membrane fluidity and cell-surface area between tumor cells and normal cells may also contribute to the selectivity of AMPs to tumor cells. Furthermore, these fundamental differences on cell membrane may, partially, explain the cell line-specific effect of PFR peptide observed in this study and other AMPs reported in previous studies^57^,^21^. Thus, deep understanding of the correlation between cell membrane compositions and PFR peptide efficacy and selectivity is critical for future clinic therapeutic application of PFR peptide.

Taken together, this study identified PFR peptide, one of antimicrobial peptides, has antitumor activity in both *in vitro* and *in vivo* models of leukemia and characterized the novel biological action of PFR peptide via necrosis induction in leukemia cells. Further investigations are therefore warranted for PFR peptide as a potential novel anti-cancer agent with important medical implications.

## Additional Information

**How to cite this article**: Lu, Y. *et al.* PFR peptide, one of the antimicrobial peptides identified from the derivatives of lactoferrin, induces necrosis in leukemia cells. *Sci. Rep.* **6**, 20823; doi: 10.1038/srep20823 (2016).

## Supplementary Material

Supplementary Information
