# Supplementary material for: PFR peptide, one of the antimicrobial peptides identified from the derivatives of lactoferrin, induces necrosis in leukemia cells
Source: Sci Rep. 2016 Feb 10;6:20823. doi: 10.1038/srep20823 (PMC4748414; doi:10.1038/srep20823)
Supplement: Supplementary Information [file srep20823-s1.pdf]

PFR peptide, one of the antimicrobial peptides identified from the derivatives of lactoferrin, induces necrosis in leukemia cells

Yan Lu[1,2,#], Teng-Fei Zhang[1,2,#], Yue Shi[1,2,#], Han-Wei Zhou[1,2], Qi Chen[1,2], Bu-Yun Wei[1,2], Xi Wang[3], Tian-Xin Yang[4], Y. Eugene Chinn[5], Jian Kang[6], Cai-Yun Fu[1,2,7,*]

[1]College of Life Sciences, Zhejiang Sci-Tech University, Hangzhou 310018, China.

[2]Zhejiang Provincial Key Laboratory of Silkworm Bioreactor and Biomedicine, Hangzhou 310018, China

[3]Department of Oncology, the 117th Hospital of PLA, 14 Lingyin Road, Hangzhou 310013, China.

[4]Department of Hematology, Zhejiang Province People's Hospital, Hangzhou, Zhejiang, China.

[5]Institute of Health Sciences, Chinese Academic of Sciences, Shanghai 200031, China.

[6]Cancer Signalling Laboratory, Oncogenic Signalling and Growth Control Program, Peter MacCallum Cancer Centre, St Andrews Place, East Melbourne, Victoria 8006, Australia.

[7] Key Laboratory of Cell-Based Drug and Applied Technology Development in Zhejiang Province, Hangzhou, People's Republic of China

[#]These authors contributed equally to this work.

* Corresponding author. Cai-Yun Fu, Tel.: 86-571-86843336; E-mail address: fucy03@126.com

Supplementary Figure S1

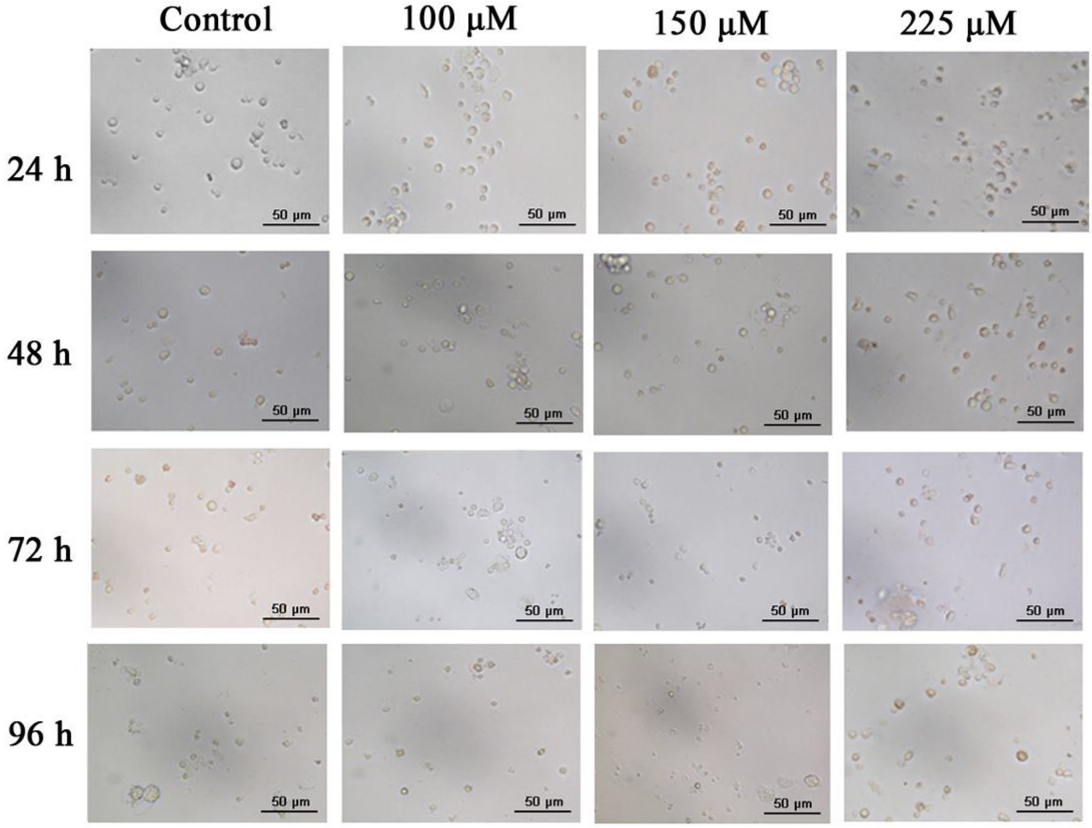

Supplementary Figure S2

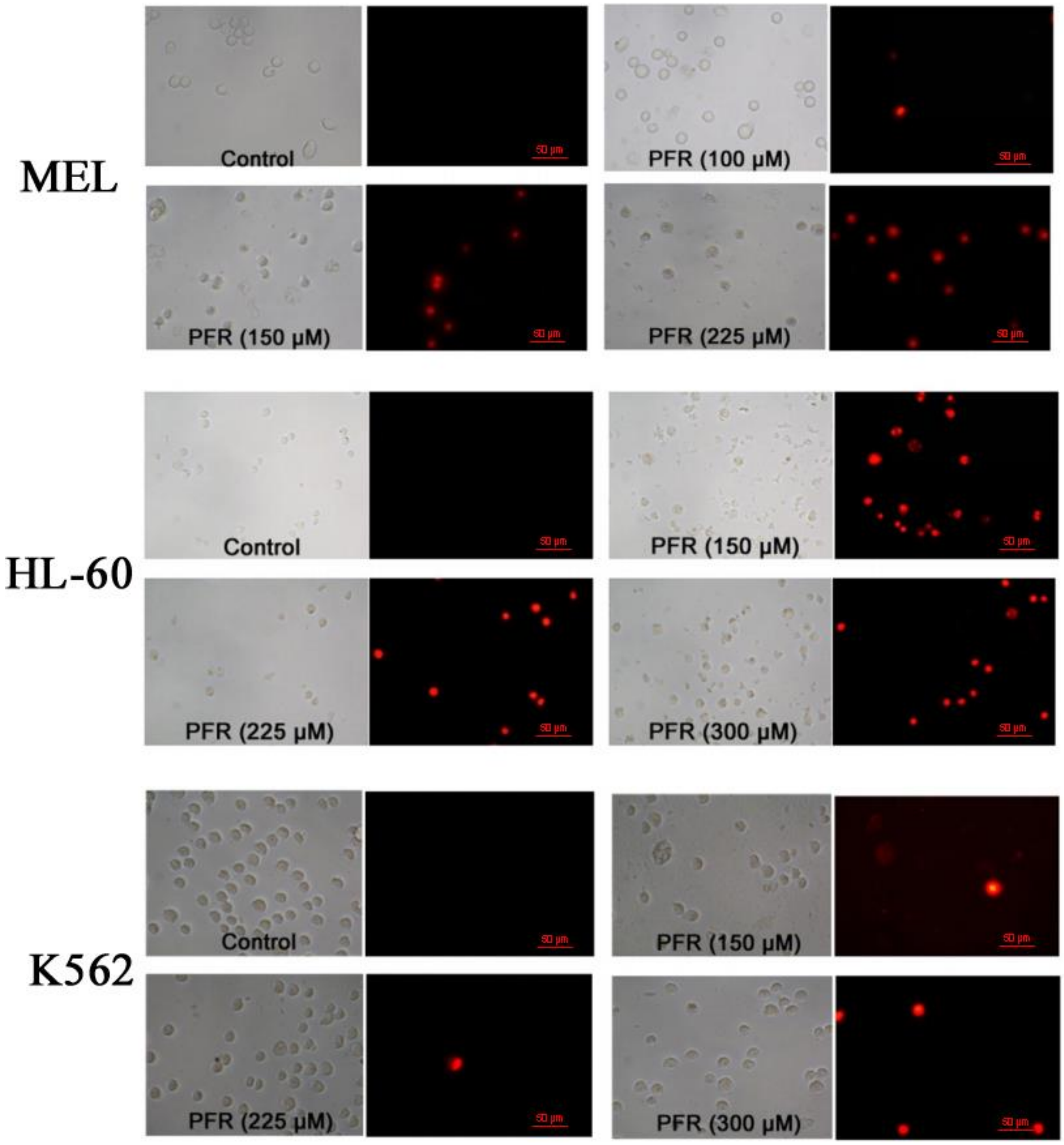

Figure Legends

Supplementary Figure S1 Changes in morphology and quantity of mouse normal bone marrow cells treated with different concentrations of PFR peptide at the indicated time points.

Supplementary Figure S2 Fluorescence microscopic analysis of PI staining in three different types of leukemia cell lines following treatment with PFR peptide for 24 hours.
